# Role of Plasma-Derived Exosomal MicroRNAs in Mediating Type 2 Diabetes Remission

**DOI:** 10.3390/nu17152450

**Published:** 2025-07-27

**Authors:** Sujing Wang, Shuxiao Shi, Xuanwei Jiang, Guangrui Yang, Deshan Wu, Kexin Li, Victor W. Zhong, Xihao Du

**Affiliations:** Department of Epidemiology and Biostatistics, School of Public Health, Shanghai Jiao Tong University School of Medicine, Shanghai 200025, China; wangsujing@sjtu.edu.cn (S.W.); hanyingyinghan@sjtu.edu.cn (S.S.); xuanweijiang@sjtu.edu.cn (X.J.); gr_yang@sjtu.edu.cn (G.Y.); xiaozuwu-99@sjtu.edu.cn (D.W.); liviking@sjtu.edu.cn (K.L.); wenze.zhong@shsmu.edu.cn (V.W.Z.)

**Keywords:** low-calorie diet, type 2 diabetes, exosome, microRNA, weight loss

## Abstract

**Objective:** This study aimed to identify plasma exosomal microRNAs (miRNAs) associated with weight loss and type 2 diabetes (T2D) remission following low-calorie diet (LCD) intervention. **Methods:** A 6-month dietary intervention targeting T2D remission was conducted among individuals with T2D. Participants underwent a 3-month intensive weight loss phase consuming LCD (815–835 kcal/day) and a 3-month weight maintenance phase (N = 32). Sixteen participants were randomly selected for characterization of plasma-derived exosomal miRNA profiles at baseline, 3 months, and 6 months using small RNA sequencing. Linear mixed-effects models were used to identify differentially expressed exosomal miRNAs between responders and non-responders. Pathway enrichment analyses were conducted using target mRNAs of differentially expressed miRNAs. Logistic regression models assessed the predictive value of differentially expressed miRNAs for T2D remission. **Results:** Among the 16 participants, 6 achieved weight loss ≥10% and 12 achieved T2D remission. Eighteen exosomal miRNAs, including miR-92b-3p, miR-495-3p, and miR-452b-5p, were significantly associated with T2D remission and weight loss. Pathway analyses revealed enrichment in PI3K-Akt pathway, FoxO signaling pathway, and insulin receptor binding. The addition of individual miRNAs including miR-15b-3p, miR-26a-5p, and miR-3913-5p to base model improved the area under the curve values by 0.02–0.08 at 3 months and by 0.02–0.06 at 6 months for T2D remission. **Conclusions:** This study identified exosomal miRNAs associated with T2D remission and weight loss following LCD intervention. Several exosomal miRNAs might serve as valuable predictors of T2D remission in response to LCD intervention.

## 1. Introduction

Type 2 diabetes (T2D) affects over 537 million people globally [1]. Since 2000, mortality rates from T2D have been increasing. Traditional pharmacological treatments for T2D, primarily focused on glucose-lowering, have had limited success in reducing its prevalence [2]. Low-calorie diet (LCD) interventions have been shown to promote weight loss and induce T2D remission [3,4,5]. However, the potential mechanisms underlying LCD intervention-induced T2D remission remain poorly understood.

Exosomes are small nano-sized vesicles, with an average diameter of 100 nm. An increasing number of studies have reported the role of exosomal microRNAs (miRNAs) in the development of T2D [6,7]. These exosomal miRNAs are involved in various metabolic pathways, including glucose homeostasis and insulin signaling [8]. Furthermore, miRNAs hold potential therapeutic implications, particularly in how dietary interventions can modulate their expression and function to benefit T2D treatment [9,10]. Despite the importance of miRNAs in the development of T2D, there remains a significant gap in understanding how LCD interventions affect exosomal miRNA profiles and their potential to mediate T2D remission.

Therefore, we conducted a 6-month clinical study to identify genome-wide changes in exosomal miRNA signatures in T2D participants following LCD intervention. Small RNA sequencing (RNA-seq) was performed to comprehensively characterize the plasma-derived exosomal miRNAs associated with weight loss and T2D remission.

## 2. Materials and Methods

### 2.1. Study Design

This clinical trial was conducted at the Shanghai Municipal Hospital of Traditional Chinese Medicine. Participants were eligible if they were aged 18 to 60 years with physician-diagnosed T2D and had a body mass index (BMI) of 24–45 kg/m^2^, hemoglobin A1c (HbA1c) level of 6.5–12.0% or <6.5% if on pharmacotherapy, and no plan to leave Shanghai within 2 years. Exclusion criteria included a diagnosis of type 1 diabetes, current using of insulin or glucagon-like peptide-1 receptor agonists, HbA1c level of >12%, cardiovascular event within the previous 6 months, weight loss >5 kg within the previous 6 months, eating disorders, an estimated glomerular filtration rate <30 mL/min/1.73 m^2^, psychiatric disorders, severe arthritis and gout, gallstone disease or known asymptomatic gallstones, participation in another clinical trial, pregnancy or consideration of pregnancy, known cancer, learning difficulties, alcoholism, and pancreatitis.

The intervention process consisted of two phases: a 3-month intensive weight loss phase followed by a 3-month weight loss maintenance phase. All participants discontinued anti-diabetic medications on the first day under medical supervision during the intervention. Standardized guidance was provided for exercise and lifestyle modifications, and the controlled intervention design minimized potential confounding effects on miRNA expression. Briefly, during the weight loss phase, participants consumed a low-calorie formula diet that provided 815–835 kcal/day. This intervention utilized commercially prepared meal replacement products with precisely controlled macronutrient composition: 43–48% carbohydrates, 27–29% protein, and 25–28% fat. The formula diet consisted of processed nutritional powders and shakes rather than whole foods, ensuring standardized nutrient delivery across all participants. During the weight loss maintenance phase, participants followed an individualized program to prevent weight regain, with supervised transition to a normal diet. Physical activity was encouraged up to 15,000 steps per day, with personalized recommendations based on individual fitness levels and health status to support sustainable weight management. A more detailed description of this intervention and its outcomes has been previously published [5].

This study was approved by the Ethics Committee of the Shanghai Municipal Hospital of Traditional Chinese Medicine (approval code: 2022SHL-KY-23-02, approval date: 26 May 2022). The trial was registered at ClinicalTrials.gov (NCT05472272). Written consent was obtained from all participants.

### 2.2. Anthropometric and Biochemical Assessments

T2D remission was defined as HbA1c level <6.5% after at least 3 months off glucose-lowering medications. Weight loss of at least 10% has been associated with a significantly higher likelihood of T2D remission [11]. Anthropometric measurements, including body weight, BMI, and body composition, were measured at baseline, 3 months, and 6 months, respectively. Body compositions included fat mass, fat mass percentage, muscle mass, muscle mass percentage, and trunk fat mass and were assessed using Tanita MC-980MA body composition analyzer. To evaluate glucose metabolism, fasting blood glucose, fasting C peptide, and HbA1c were measured. The updated homeostatic model assessment (HOMA2) was used to estimate insulin sensitivity (HOMA2-IR) and beta-cell function (HOMA2-%β), calculated based on fasting C peptide and glucose levels.

### 2.3. Blood Collection, Exosome Purification, and Exosome Characterization

Among 32 participants who completed the intervention, 16 participants were randomly selected for exosomal RNA-seq analysis. There was no significant baseline difference between the chosen participants and those not selected for exosomal small RNA-seq analysis (Table S1). Blood samples were collected at baseline, 3 months, and 6 months after an overnight fast (10–12 h). The three assessment timepoints were strategically chosen to capture key metabolic transition phases: early adaptation (3 months) and sustained response (6 months). Plasma was promptly separated from collected samples in the EDTA tube via centrifugation at 3000 rpm for 10 min at 4 °C. Subsequently, exosomes were isolated using differential ultracentrifugation. Briefly, the samples underwent sequential centrifugation steps: first at 2000× *g* for 30 min at 4 °C to remove cells and debris, followed by centrifugation at 10,000× *g* for 45 min at 4 °C to eliminate larger vesicles. The resulting supernatant was filtered through 0.45 μm filters and subjected to ultracentrifugation at 100,000× *g* for 70 min at 4 °C using an ultracentrifuge rotor (Hitachi, Tokyo, Japan). The pellet was resuspended in 10 mL of pre-chilled 1×PBS and underwent a second ultracentrifugation wash at 100,000× *g* for 70 min at 4 °C. The final exosome pellet was resuspended in 400 μL of pre-chilled 1×PBS, with aliquots allocated for electron microscopy verification, particle size analysis, and protein quantification. The exosome morphology was measured using transmission electron microscopy (Hitachi, Tokyo, Japan). The concentration and size distribution of exosomes were analyzed using nanoparticle tracking analysis with a NanoFCM (NanoFCM Inc., Xiamen, China). The western blot was performed to analyze the biomarker of exosome, including TSG101, CD9, and calnexin.

### 2.4. Small RNA Isolation and Sequencing

Total RNA was extracted from the purified exosomes using the Qiagen exoRNeasy Maxi Kit (Qiagen, Hilden, Germany). RNA quality and integrity were assessed using the NanoPhotometer^®^ spectrophotometer (IMPLEN, Westlake Village, CA, USA) and Agilent Bioanalyzer 2100 system (Agilent Technologies, Santa Clara, CA, USA), with samples having RNA integrity number (RIN) score >7 used for further processing. Quantification was performed using the Qubit^®^ RNA Assay Kit in a Qubit^®^ 2.0 Fluorometer (Life Technologies, Carlsbad, CA, USA), ensuring sufficient RNA input for downstream analyses. Small RNA libraries were constructed from 10 ng total RNA per sample, using the NEBNext^®^ Multiplex Small RNA Library Prep Set for Illumina^®^ (NEB, Ipswich, CA, USA). The library preparations were sequenced on an Illumina Novaseq platform, and 50bp single-end reads were generated. Briefly, the raw sequencing data in FASTQ format underwent processing using custom Perl (version 5.32.1) and Python (version 3.9.0) scripts. Starting with high-precision mapping of small RNA tags to the human genome using Bowtie [12]. Subsequent steps involved identifying known miRNAs using miRBase 20.0, coupled with mirdeep2 (version 2.0.0.8) and srna-tools-cli (version 1.0.0) for structural visualization and analysis. To focus on miRNAs, we removed interfering RNA types like rRNAs and tRNAs using databases such as Repeat Masker and Rfam. Novel miRNAs were predicted through analysis of unannotated tags, looking for typical miRNA precursor characteristics with tools like miREvo and mirdeep2 [13,14].

### 2.5. Statistical Analysis

Data are expressed as mean (standard deviation) for continuous variables or frequencies and percentages for categorical variables. The Student *t*-test (for continuous variables) and the chi-squared test (for categorical variables) were used to test differences in characteristics between groups. MiRNAs with read counts of zero or missing in more than 50% of the samples were excluded to focus on mature and consistently expressed miRNAs. All miRNA read counts were normalized using counts per million and then applied with log2 transformation. Orthogonal partial least squares discriminant analysis (OPLS-DA) was performed to examine the miRNA differences between groups using the “ropls” package.

Generalized linear mixed models were employed using the glmer function from the “lme4” package to identify differentially expressed miRNAs between participants with and without T2D remission and participants with significant weight loss (≥10%) or not. The models were adjusted for age (years), sex (men and women), duration of T2D (years), and BMI (kg/m^2^) at baseline as fixed effects, and each patient was treated as a random effect. *p* values were adjusted using the Benjamini-Hochberg method to control the false discovery rate (FDR), and miRNAs were considered differentially expressed if the FDR-adjusted *p* value was <0.05. Target prediction was performed to explore the potential regulatory impacts of differentially expressed miRNAs using miRTargetLink 2.0, with only target mRNAs regulated by at least two miRNAs retained for further analysis [15].

To understand the biological functions and pathways potentially influenced by the differentially expressed miRNAs, enrichment analyses were conducted using the Kyoto Encyclopedia of Genes and Genomes (KEGG) database and Gene Ontology (GO). The GO included molecular function, cellular component, and biological process. The top 20 significantly enriched pathways of KEGG were presented based on FDR-adjusted *p* < 0.05.

Spearman correlations between differentially expressed miRNAs and phenotypes, including body composition and blood glucose levels, were estimated. For categorical variables, Mann-Whitney U tests were used to compare miRNA expression between participants with and without family history of T2D. Logistic regression models were constructed to evaluate the performance of differentially expressed miRNAs for predicting T2D remission at 3 and 6 months. The base model included age, sex, duration of T2D, and baseline BMI. The expression level of each identified miRNA at baseline was then added to the model to assess its impact on predictive accuracy. The predictive performance was assessed by area under the curve (AUC) of the receiver operating characteristic (ROC) curve. The ROC curves with improved AUC values compared with the basic model were plotted. All analyses were performed using R version 4.4.1.

## 3. Results

### 3.1. Characteristics of the Participants

After a 6-month intervention, among the 16 participants, 12 (75.0%) achieved T2D remission and 6 (37.5%) achieved weight loss ≥10%. Participants who achieved these goals were characterized by lower age, shorter T2D duration, lower HbA1c, lower fasting blood glucose, lower body fat percentage, and higher body muscle percentage compared to those who did not (Table 1). Similar characteristics were observed at the 3-month follow-up, where 11 (68.8%) participants achieved T2D remission and 9 (56.3%) participants achieved weight loss ≥10% (Table S2).

### 3.2. Characterization of Plasma-Derived Exosomes and Small RNA Quality Assessment

Transmission electron microscopy revealed cup-shaped, rounded vesicle-like structures with a double membrane (Figure S1A). Nanoparticle tracking analysis revealed a size distribution of spherical nanoparticles ranging from 30 to 150 nm, with a prominent peak centered at approximately 80 nm (Figure S1B). The size distribution aligned with the typical characteristics of exosomes. Western blot analysis showed that TSG101 and CD9 were significantly enriched in plasma exosomes, while calnexin was absent (Figure S1C). The small RNA-seq data exhibited consistently high quality, with average Q30 and Q20 scores exceeding 97% and 99% respectively (Table S3). Stable GC content of around 53.5% ensured accurate sequencing interpretations.

### 3.3. Differentially Expressed Plasma-Derived Exosomal miRNAs

**Figure 1:**
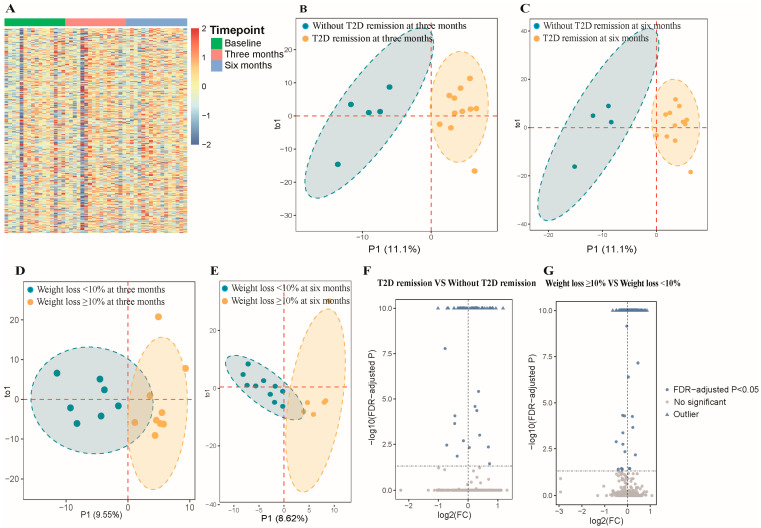
Comprehensive analysis of microRNAs from plasma exosomes in participants. Abbreviations: FC, fold change; FDR, false discovery rate; T2D, type 2 diabetes. (**A**) Heatmap of 472 microRNAs (miRNAs) expression levels across different timepoints (baseline, three months, six months). (**B**) Orthogonal partial least squares discriminant analysis (OPLS-DA) plot for remission vs. non-remission groups at three months based on expression of miRNAs at baseline. Blue and orange areas represent 95% confidence ellipses for T2D remission and non-remission groups, respectively. Dashed lines indicate the axes of principal components. (**C**) OPLS-DA plot for remission vs. non-remission groups at six months based on expression of miRNAs at baseline. Blue and orange areas represent 95% confidence ellipses for T2D remission and non-remission groups, respectively. Dashed lines indicate the axes of principal components. (**D**) OPLS-DA plot for weight loss ≥10% vs. <10% groups at three months based on expression of miRNAs at baseline. Blue and orange areas represent 95% confidence ellipses for weight loss ≥10% and <10% groups, respectively. Dashed lines indicate the axes of principal components. (**E**) OPLS-DA plot for weight loss ≥10% vs. <10% groups at six months based on expression of miRNAs at baseline. Blue and orange areas represent 95% confidence ellipses for weight loss ≥10% and <10% groups, respectively. Dashed lines indicate the axes of principal components. (**F**) Volcano plot for differential expression analysis (remission vs. non-remission). The log2 (FC) was calculated as the ratio of the average expression level of each miRNA in the remission group to the average expression level of each miRNA in the non-remission group. The −log10 (FDR-adjusted *p*) was calculated from the FDR-adjusted *p* obtained in the linear mixed model, where values above 10 were defined as outliers. Blue dots represent significantly differentially expressed miRNAs (FDR-adjusted *p* < 0.05). Gray dots represent non-significantly expressed miRNAs. Dashed lines indicate significance thresholds (horizontal: FDR-adjusted *p* = 0.05; vertical: log2 FC = 0). (**G**) Volcano plot for differential expression analysis (weight loss ≥10% vs. <10%). The log2 (FC) was calculated as the ratio of the average expression level of each miRNA in the weight loss ≥10% group to the average expression level of each miRNA in the weight loss <10% group. Blue dots represent significantly differentially expressed miRNAs (FDR-adjusted *p* < 0.05). Gray dots represent non-significantly expressed miRNAs. Dashed lines indicate significance thresholds (horizontal: FDR-adjusted *p* = 0.05; vertical: log2 FC = 0).

**Figure 2:**
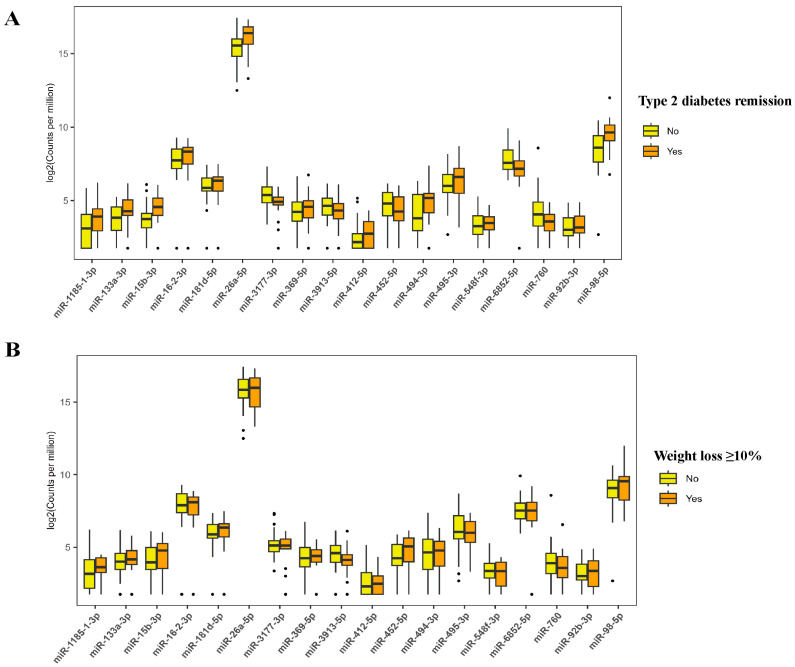
Expression level of microRNAs that are differentially expressed in participants with remission vs. non-remission of type 2 diabetes and weight loss ≥10% vs. <10% groups. The boxplots show microRNA expression levels where the box represents the interquartile range (25–75th percentiles), the horizontal line indicates the median, whiskers extend to 1.5 times the interquartile range, and black dots represent outliers (individual data points that fall outside 1.5 times the interquartile range from the edges of the box). (**A**) Expression levels of microRNAs differentially expressed between participants who achieved type 2 diabetes remission (orange boxes) versus those who did not achieve remission (yellow boxes). (**B**) Expression levels of microRNAs differentially expressed between participants who achieved weight loss ≥10% (orange boxes) versus those who achieved weight loss <10% (yellow boxes).

A total of 1434 mature miRNAs were initially identified from sequencing 48 samples. After data preprocessing, 472 miRNAs were retained for further analysis. Figure 1A shows the overall expression of these miRNAs. OPLS-DA analysis differentiated subgroups based on remission status and significant weight loss at 3 and 6 months as indicated by distinct clustering according to baseline miRNA expression (Figure 1B–E). Generalized linear mixed models identified 63 differentially expressed miRNAs among participants who achieved T2D remission versus those who did not, and 129 miRNAs were significantly different between those who achieved weight loss ≥10% and those who did not (FDR-adjusted *p* < 0.05; Figure 1F,G). The expression levels of the identified miRNAs are shown in Figure S2A,B. Figure S2C shows 18 miRNAs that were common to both groups, such as miR-494-3p, miR-15b-3p, miR-92b-3p, miR-26a-5p, and miR-3913-5p. Tables S4 and S5 present the coefficient and FDR-adjusted *p* values of these 18 miRNAs. The expression level of miR-26a-5p and miR-15b-3p was higher in the remission and successful weight loss groups compared to those in the non-remission and unsuccessful weight loss groups (Figure 2).

**Figure 3:**
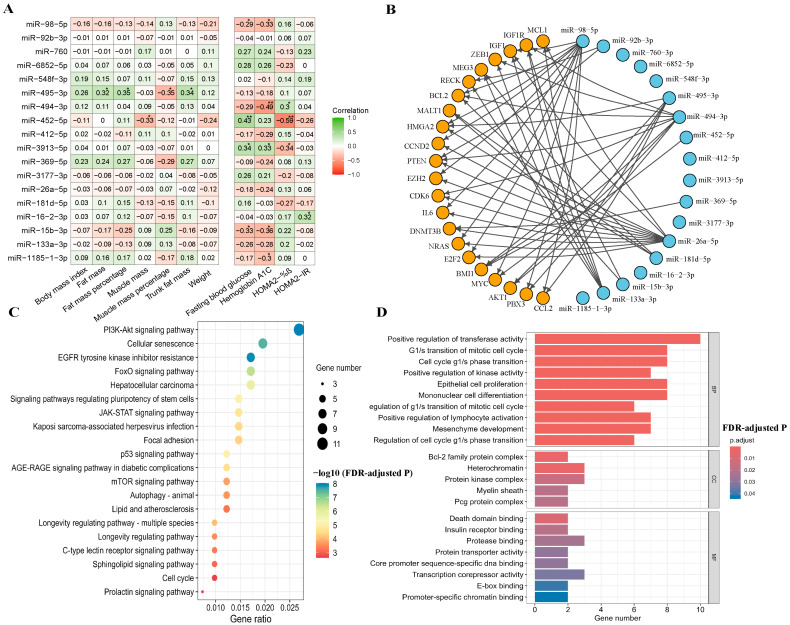
Correlation, interaction network, and enrichment analysis. Abbreviations: %β, beta-cell function; BP, biological process; CC, cellular component; FDR, false discovery rate; HOMA2, Homeostasis Model Assessment 2; IR, insulin resistance; MF, molecular function. (**A**) Correlation of significant microRNAs with anthropometric and biochemical measurements. * *p* < 0.05, ** *p* < 0.01. (**B**) A network of interactions between 18 microRNAs and their target genes. These microRNAs were connected by lines to various target genes (mRNAs) indicated by orange circles. Arrows indicate the direction of regulatory interactions from miRNAs to their target mRNAs. (**C**) A bubble chart of Kyoto Encyclopedia of Genes and Genomes (KEGG) pathway analysis for the target genes of the miRNAs from Panel (**B**). Bubble color indicates the significance of pathway enrichment (−log10 FDR-adjusted *p*), and bubble size reflects the number of genes involved in each pathway. (**D**) A bar chart of Gene Ontology (GO) analysis results. The bars were colored representing different levels of statistical significance (FDR-adjusted *p*), with bar length indicating the number of genes associated with each term.

Among 18 differentially expressed miRNAs, 8 of them were significantly correlated with anthropometric and biochemical measurements. For example, miR-495-3p was positively correlated with fat mass, fat percentage, and trunk fat mass, but was negatively correlated with muscle percentage (Figure 3A). MiR-452-5p showed negative correlations with muscle mass and HOMA2-%β, while showing a positive correlation with fasting blood glucose. MiR-494-3p showed a significant negative correlation with HbA1c and a positive correlation with HOMA2-%β. MiR-15b-3p and miR-98-5p showed negative correlations with HbA1c and fasting blood glucose, whereas miR-3913-5p was positively correlated with these measures. Only miR-16-2-3p was found to be positively correlated with HOMA2-IR. Participants with a family history of T2D showed significantly lower expression of miR-495-3p compared to those without family history (5.42 ± 1.20 vs. 6.61 ± 1.31, FDR-adjusted *p* = 0.02) (Table S6).

### 3.4. Pathway Enrichment Analysis

The network diagram shows 22 targeted mRNAs (such as HMGA2, BMI1, BCL2, IGF1, AKT1, PTEN, and IGF1R) regulated by the 18 identified miRNAs (Figure 3B and Table S7). The top 20 significantly enriched KEGG pathways included PI3K-Akt signaling pathway, cellular senescence, EGFR tyrosine inhibitor resistance, and FoxO signaling pathway (Figure 3C and Table S8). GO analysis identified 23 significantly enriched terms, including 10 biological processes, 5 cellular components, and 8 molecular functions. These enriched terms included positive regulation of transferase activity, G1/S transition of the mitotic cell cycle, and insulin receptor binding (Figure 3D and Table S9).

### 3.5. Receiver Operating Characteristic Curve Analysis

**Figure 4:**
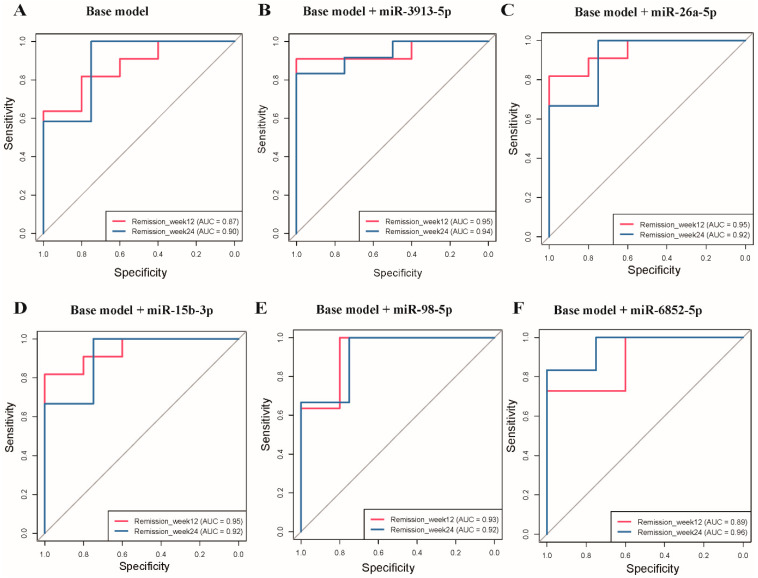
Receiver operating characteristic (ROC) curves for type 2 diabetes remission prediction at week 12 and week 24. Base model including age, sex, duration of type 2 diabetes, and body mass index at baseline. The diagonal gray line in each panel represents the line of no discrimination (random chance, AUC = 0.5). (**A**) ROC curves for the base model alone at week 12 (AUC = 0.87) and week 24 (AUC = 0.90). (**B**) Base model + miR-3913-5p showing improved performance at week 12 (AUC = 0.95) and week 24 (AUC = 0.94). (**C**) Base model + miR-26a-5p demonstrating enhanced accuracy at week 12 (AUC = 0.95) and week 24 (AUC = 0.92). (**D**) Base model + miR-15b-3p showing improved discrimination at week 12 (AUC = 0.95) and week 24 (AUC = 0.92). (**E**) Base model + miR-98-5p illustrating enhanced performance at week 12 (AUC = 0.93) and week 24 (AUC = 0.92). (**F**) Base model + miR-6852-5p demonstrating improved accuracy at week 12 (AUC = 0.89) and week 24 (AUC = 0.96).

The base model shows good predictive accuracy with AUC values of 0.87 and 0.90 for remission at 3 and 6 months, respectively (Figure 4A). The addition of individual miRNAs improved the predictive performance by 0.02–0.08 at 3 months (AUC: 0.89–0.95) and by 0.02–0.06 at 6 months (AUC: 0.92–0.96). For example, miR-3913-5p enhanced the AUC to 0.95 and 0.94 at 3 and 6 months (Figure 4B), and miR-26a-5p increased the AUC to 0.95 and 0.92 (Figure 4C). Similar improvements were observed with miR-15b-3p (AUC = 0.95 and 0.92; Figure 4D), miR-98-5p (AUC = 0.93 and 0.92; Figure 4E), and miR-6852-5p (AUC = 0.89 and 0.96; Figure 4F), all demonstrating strong predictive capabilities for T2D remission at both time points.

## 4. Discussion

To our knowledge, this is the first study using small RNA-seq to investigate the role of exosomal miRNAs in mediating T2D remission after LCD intervention. The differentially expressed miRNAs were mainly involved in critical processes such as PI3K-Akt pathway and insulin receptor binding. Several differentially expressed miRNAs, such as miR-495-3p and miR-452-5p, were correlated with fat mass, muscle mass, and fasting blood glucose. Furthermore, miRNAs such as miR-92b-3p, miR-15b-3p, and miR-26a-5p were identified as valuable predictors of T2D remission.

The differentially expressed miRNA signatures provided insights into the role of exosomal miRNAs on T2D remission through weight loss. We focused specifically on exosomal miRNAs due to their enhanced stability and role as functional intercellular messengers, which provides greater mechanistic relevance compared to total plasma miRNAs that include degraded species. Our findings identified several exosomal miRNAs, including miR-92b-3p, miR-26a-5p, miR-98-5p, and miR-133a-3p, that play important roles in T2D remission, which were also reported by previous studies. For example, an animal study suggested the anti-diabetic effects of miR-92b-3p in T2D mice via regulation of the miR-92b-3p/EGR1 axis, which improved insulin resistance and pancreatic function [16]. Upregulation of miR-26a-5p has been linked to inflammation and insulin resistance [17]. MiR-98-5p and miR-133a-3p, both associated with obesity and lipid metabolism, may also contribute to T2D remission [18,19]. Additionally, other differentially expressed exosomal miRNAs including miR-494-3p, miR-15b-3p, miR-3913-5p, and miR-412-5p were also identified in this study. Although these miRNAs were not directly associated with obesity or diabetes, growing evidence suggests that they play critical roles in the regulation of key metabolic processes. For instance, miR-494-3p has been linked to the modulation of mitochondrial biogenesis and function, which are essential for maintaining cellular energy homeostasis and preventing metabolic disorders [20,21]. Similarly, miR-15b-3p is involved in the regulation of apoptosis and oxidative stress, processes that are often dysregulated in metabolic diseases [22]. MiR-412-5p and miR-3913-5p, though less studied, have been implicated in pathways influencing inflammatory responses and lipid transport, both of which are critical factors in the progression of obesity-related complications [23,24]. Collectively, these miRNAs contribute to a better understanding of the complex molecular mechanisms underlying metabolic diseases and may represent novel therapeutic targets for T2D.

Functional and pathway analyses suggested that differentially expressed miRNAs were mainly enriched in the PI3K-Akt signaling pathway, FoxO signaling pathway, and insulin receptor binding. The PI3K-Akt pathway plays a critical role in regulating glucose metabolism and insulin sensitivity, and its dysregulation is commonly observed in diabetic states [25]. An LCD intervention may restore proper function to this pathway, thereby improving insulin responsiveness. Similarly, the FoxO signaling pathway, which governs oxidative stress and glucose homeostasis, may benefit from caloric restriction, leading to improved glycemic control and reduced oxidative damage—both essential for maintaining metabolic health during T2D remission [26,27]. Furthermore, the enhancement of insulin receptor binding affinity strengthens the insulin signaling cascade, thereby improving glucose uptake and cellular metabolic responses [28]. These enriched pathways collectively contribute to the therapeutic effects of an LCD intervention in promoting T2D remission.

The correlations between exosomal miRNAs and various anthropometric and biochemical measurements provide valuable insights into their potential roles in metabolic regulation. For instance, the correlation of miR-452-5p with glycemic parameters and β-cell function is aligned with its established role in regulating branched-chain α-keto acid dehydrogenase-β gene expression, which influences pancreatic β-cell function through branched-chain amino acid (BCAA) metabolism [29]. BCAAs are known to modulate insulin secretion and β-cell proliferation, and the disruption of their metabolism has been linked to insulin resistance in T2D [30]. The correlation of miR-495-3p with body composition parameters aligns with its role in regulating key metabolic genes. MiR-495-3p may target HMGA2, AKT1, and CCL2, which are critically involved in adipocyte differentiation and lipid metabolism. For example, AKT1 played a particularly important role in insulin-mediated metabolic actions and β-cell mass regulation [31]. The regulatory function of miR-495-3p in these pathways aligns with its documented involvement in various biological processes, including development, inflammation, and immunological responses [32]. The dysregulation of miR-495-3p may contribute to the development of obesity and T2D by impairing the balance between adipogenesis and lipolysis [33]. In addition, the observed correlations for miR-494-3p, miR-15b-3p, miR-98-5p, and miR-3913-5p with various metabolic parameters, particularly those related to glycemic control, highlight their potential roles in regulating key pathways such as glucose uptake, insulin sensitivity, and inflammatory responses. While the potential roles of these miRNAs in metabolic regulation are promising, their mechanistic functions remain to be fully elucidated. It is essential to explore their interactions with target genes in more depth, especially within the context of T2D and obesity. Further studies are needed to establish causal relationships, identify novel therapeutic targets, and better understand how these miRNAs contribute to the pathophysiology of metabolic diseases. Additionally, as the roles of exosomal miRNAs in intercellular communication and tissue-specific responses are still being explored [34], understanding how these miRNAs influence distant organs, such as the liver and muscle, may provide crucial insights into systemic metabolic regulation.

MiRNAs have emerged as important biomarkers for predicting both the onset and progression of T2D [35,36]. Recent studies have demonstrated their utility in predicting treatment outcomes [37]. Our study identified three exosomal miRNAs (miR-15b-3p, miR-26a-5p, and miR-3913-5p) that might serve as predictive markers for T2D remission. The predictive capability of these miRNAs is underscored by their involvement in critical metabolic pathways, particularly PI3K-Akt and FoxO signaling, both of which are essential for regulating glucose homeostasis and insulin sensitivity [38]. These pathways have been well-documented in the context of T2D, as they govern cellular responses to insulin and glucose, thus influencing metabolic balance [25]. Furthermore, the observed correlations between these miRNAs and various metabolic parameters, including the correlation of miR-3913-5p with glycemic measures, substantiate their role as biomarkers for metabolic responsiveness. Previous studies have shown that alterations in miRNA expression profiles often correlate with the pathophysiology of T2D, particularly through their regulation of genes involved in glucose metabolism and insulin signaling [39]. These miRNAs may also serve as non-invasive diagnostic tools, facilitating early identification of individuals at risk for T2D or its remission.

This study has several limitations. First, the small sample size limits statistical power and warrants caution when interpreting results. Second, the absence of external validation in independent populations restricts the generalizability of our miRNA biomarkers, and replication studies are essential to establish their clinical utility. Third, all participants were of Chinese ethnicity, which may limit applicability to other ethnic populations given potential differences in miRNA expression profiles and dietary intervention responses. Fourth, the binary classification of responders may oversimplify patient heterogeneity, as participants likely varied in diabetes duration, insulin sensitivity, and medication history, potentially confounding miRNA associations. Fifth, the cellular origin of circulating exosomes was not determined, restricting our ability to infer tissue-specific contributions to the observed miRNA signatures. Finally, the lack of in vitro mechanistic studies limited our ability to establish causal relationships between identified miRNAs and their associated biological pathways.

Future research should prioritize large-scale validation studies across diverse populations to establish the clinical utility of exosomal miRNA biomarkers for predicting T2D remission. High-frequency temporal sampling and multi-omics integration could elucidate the dynamic mechanisms linking miRNA changes to metabolic outcomes, while characterization of exosome cellular origins would provide insights into tissue-specific regulation. Development of personalized prediction models incorporating miRNA signatures with clinical and genetic factors through machine learning could enable precision medicine approaches for tailored diabetes interventions. Long-term studies examining the sustainability of miRNA changes and their relationship to durable metabolic outcomes, coupled with health economic evaluations, will be essential for translating these biomarker discoveries into routine clinical practice for diabetes management.

## 5. Conclusions

In conclusion, our study revealed differential expression of plasma-derived exosomal miRNAs between responders and non-responders to the LCD intervention. These findings enhance our understanding of the molecular mechanisms involved in T2D remission and suggest that exosomal miRNAs may serve as potential biomarkers for predicting T2D remission. Further research is required to validate these miRNAs and elucidate their functional roles in T2D pathophysiology.

## Figures and Tables

**Table 1:** Characteristics of participants with small RNA sequencing at baseline and six months.

Characteristic	Baseline	Six Months				
	Overall	Overall	Weight Loss ≥ 10%	Weight Loss < 10%	Type 2 Diabetes Remission	Without Type 2 Diabetes Remission
N	16	16	6	10	12	4
Age, mean (SD), year	37.3 (8.0)	37.3 (8.0)	40.7 (4.6)	35.3 (9.1)	37.0 (7.6)	38.3 (10.5)
Men, n (%)	12 (75.0)	12 (75.0)	4 (66.6)	8 (80.0)	11 (91.7)	1 (25.0)
Duration of T2D, mean (SD), year	2.5 (2.1)	2.5 (2.1)	1.8 (1.7)	2.9 (2.3)	2.2 (2.4)	3.5 (0.8)
Family history of T2D, n (%)	8 (50.0)	8 (50.0)	5 (83.3)	3 (30.0)	6 (50.0)	2 (50.0)
Body weight, mean (SD), kg	92.4 (26.7)	82.4 (24.1)	70.3 (11.3)	89.6 (27.2)	84.8 (26.6)	75.0 (14.5)
Body mass index, mean (SD), kg/m^2^	31.2 (6.9)	27.8 (6.7)	24.1 (2.5)	30.0 (7.6)	28.1 (7.5)	26.9 (4.4)
Body fat mass, mean (SD), kg	32.3 (16.9)	23.4 (14.7)	15.8 (5.9)	28.5 (16.9)	23.8 (16.3)	22.0 (6.3)
Body fat percentage, %	33.6 (9.8)	26.9 (9.6)	22.9 (8.8)	29.5 (9.6)	25.9 (9.9)	30.7 (8.5)
Body muscle mass, mean (SD), kg	55.9 (13.3)	55.1 (12.6)	51.2 (12.2)	57.7 (12.8)	57.2 (11.9)	46.6 (14.0)
Body muscle percentage, %	61.9 (9.3)	68.4 (9.7)	72.6 (9.9)	65.6 (8.9)	69.5 (10.1)	63.9 (7.4)
Trunk fat mass, mean (SD), kg	18.0 (9.1)	12.9 (8.4)	8.4 (3.9)	15.9 (9.4)	13.3 (9.3)	11.5 (3.5)
Fasting blood glucose, mean (SD), mmol/L	7.2 (1.7)	6.2 (1.9)	5.3 (0.7)	6.8 (2.2)	5.5 (0.7)	8.6 (2.6)
Hemoglobin A1c, mean (SD), %	8.0 (1.8)	6.2 (1.0)	5.8 (0.7)	6.5 (1.1)	5.8 (0.4)	7.6 (1.0)
HOMA2-IR	2.5 (1.0)	2.0 (0.8)	1.2 (0.3)	2.4 (0.5)	2.0 (0.8)	1.8 (0.9)
HOMA2-%β	100.7 (51.2)	108.7 (49.8)	101.9 (36.7)	112.7 (57.8)	127.7 (41.3)	51.6 (18.9)

Abbreviations: %β, beta-cell function, HOMA2, Homeostasis Model Assessment 2; IR, insulin resistance; SD, standard deviation.

## Data Availability

The data presented in this study are available on request from the corresponding author due to privacy and ethical restrictions.

## Supplementary Materials

The following supporting information can be downloaded at: https://www.mdpi.com/article/10.3390/nu17152450/s1, Figure S1: Isolation and characterization of plasma-derived exosome.; Figure S2: Differential expression of microRNAs in participants with remission vs. non-remission of type 2 diabetes and weight loss ≥10% vs. <10% groups; Table S1: Baseline characteristics of participants with selection for sequencing miRNA or not; Table S2: Characteristics of participants with small RNA sequencing at three months; Table S3: The detected reads and quality control of sequencing; Table S4: Differentially expressed exosomal microRNAs in remission vs. non-remission of type 2 diabetes; Table S5: Differentially expressed miRNAs in weight loss ≥10% vs. <10% groups; Table S6: Differential miRNA expression profiles in individuals with and without family history of type 2 diabetes; Table S7: Differentially expressed miRNAs targets that strong validated; Table S8: Differentially expressed miRNAs targets in Kyoto Encyclopedia of Genes and Genomes; Table S9: Differentially expressed miRNAs targets in Gene Ontology.
