# Supplementary material for: Role of Plasma-Derived Exosomal MicroRNAs in Mediating Type 2 Diabetes Remission
Source: Nutrients. 2025 Jul 27;17(15):2450. doi: 10.3390/nu17152450 (PMC12348826; doi:10.3390/nu17152450)
Supplement: Supplementary file 1 [file nutrients-17-02450-s001.zip › nutrients-3755919-supplementary.pdf]

Role of Plasma-Derived Exosomal MicroRNAs in Mediating Type 2 Diabetes Remission

Supplementary Materials

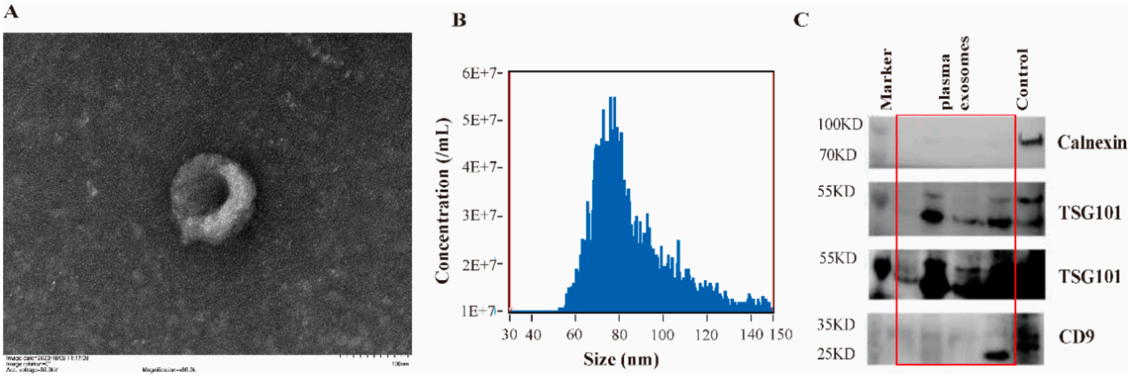

Supplementary Figure S1. *Isolation and characterization of plasma-derived exosomes.*

(A) Representative transmission electron microscopy images, scale bar = 100 nm.

(B) Representative graphs of nanoparticle tracking analysis.

(C) Representative immunoblot images of exosomal markers of tumor susceptibility gene 101 (TSG101), CD9, and calnexin.

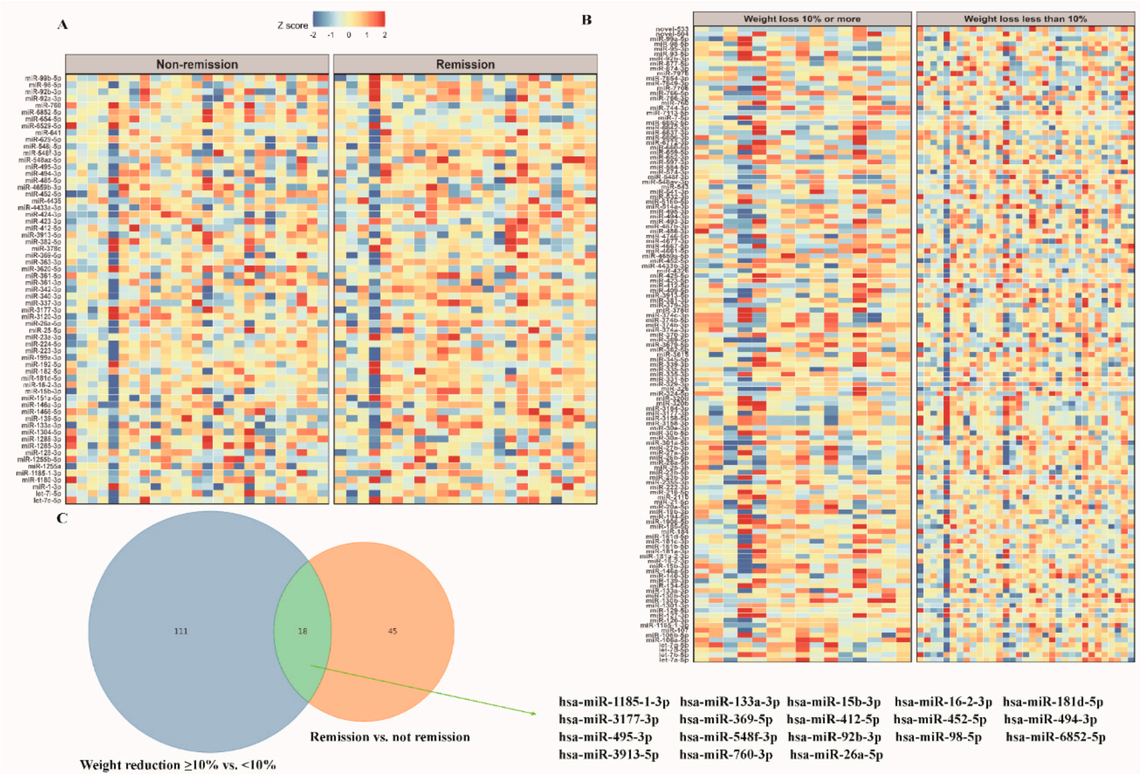

Supplementary Figure S2*. Differential expression of microRNAs in participants with remission* vs. *non-remission of type 2 diabetes and weight loss ≥10%* vs. *<10% groups.*

(A) Heatmap of differentially expressed microRNAs (miRNAs) in remission vs. non-remission groups.–

(B) Heatmap of differentially expressed miRNAs in weight loss ≥10% vs. <10% groups.

(C) Venn diagram of differentially expressed miRNAs in weight loss ≥10% or not and remission or not groups. The intersection (orange) showed miRNAs common to both conditions, with 18 miRNAs overlapping. The list on the right named the miRNAs found in the overlapping region.

Supplementary Table S1. *Baseline characteristics of participants with selection for sequencing miRNA or not.*

	**All**	**Selection**	**Not selection**	***P* value[a]**
N	32	16	16	
Age, mean (SD), year	39.8 (8.9)	42.3 (9.4)	37.3 (8.0)	0.12
Men, n (%)	21 (65.6)	9 (56.2)	12 (75.0)	0.53
Education, n (%)				0.18
< Bachelor's degree	11 (34.3)	6 (37.5)	5 (31.2)	
Bachelor's degree	17 (53.1)	9 (56.2)	8 (50.0)	
> Bachelor's degree	4 (12.5)	1 (6.2)	3 (18.8)	
No. of glucose-lowering drugs, n (%)				0.07
0	10 (31.2)	6 (37.5)	4 (25.0)	
1	8 (25.0)	1 (6.2)	7 (43.8)	
≥2	14 (43.7)	9 (56.3)	5 (31.2)	
Duration of T2D, mean (SD), year	2.5 (2.2)	2.6 (2.4)	2.5 (2.1)	0.92
Family history of T2D, n (%)	18 (56.2)	10 (62.5)	8 (50.0)	0.72
Body weight, mean (SD), kg	85.9 (21.2)	79.5 (11.5)	92.4 (26.7)	0.09
Body mass index, mean (SD), kg/m^2	29.8 (5.4)	28.4 (2.9)	31.2 (6.9)	0.15
Fasting glucose, mean (SD), mmol/L	7.4 (2.4)	7.5 (2.9)	7.2 (1.7)	0.69
Hemoglobin A1c, mean (SD), %	8.0 (1.8)	8.0 (1.9)	8.0 (1.8)	0.97
HOMA2-IR	2.6 (1.3)	2.7 (1.5)	2.5 (1.0)	0.65
HOMA2-%β	101.5 (53.4)	102.2 (57.1)	100.7 (51.2)	0.94

Abbreviations: HOMA2, Homeostasis Model Assessment 2; IR, insulin resistance; SD, standard deviation; %β, beta-cell function.

[a]Differences between groups were assessed using the t-test or chi-square test where relevant.

Supplementary Table S2. *Characteristics of participants with small RNA sequencing at three months.*

Characteristic	Overall	Weight loss ≥10%	Weight loss <10%	Type 2 diabetes remission	Without type 2 diabetes remission
N	16	9	7	11	5
Age, mean (SD), year	37.3 (8.0)	38.3 (9.4)	36.0 (6.2)	38.0 (7.0)	35.8 (10.6)
Men, n (%)	12 (75.0)	6 (66.6)	6 (71.4)	10 (90.9)	2 (40.0)
Duration of T2D, mean (SD), year	2.5 (2.1)	2.7 (2.7)	2.3 (1.3)	2.3 (2.4)	2.9 (1.5)
Family history of T2D, n (%)	8 (50.0)	6 (66.7)	2 (28.6)	6 (54.5)	2 (40.0)
Body weight, mean (SD), kg	80.7 (23.9)	76.1 (25.0)	86.6 (22.9)	78.3 (21.3)	86.0 (31.0)
Body mass index, mean (SD), kg/m^2	27.2 (6.7)	25.3 (5.7)	29.7 (7.5)	26.4 (6.8)	28.9 (6.8)
Body fat mass, mean (SD), kg	24.2 (13.8)	21.8 (12.5)	27.3 (15.9)	21.1 (13.5)	31.0 (13.3)
Body fat percentage, %	28.7 (9.7)	27.9 (9.0)	29.7 (11.1)	25.4 (9.2)	35.9 (6.7)
Body muscle mass, mean (SD), kg	53.2 (12.2)	50.9 (14.0)	56.0 (9.7)	53.8 (9.6)	51.7 (17.9)
Body muscle percentage, %	67.2 (9.1)	67.8 (8.6)	66.5 (10.5)	70.3 (8.5)	60.5 (7.0)
Trunk fat mass, mean (SD), kg	13.5 (7.9)	12.2 (7.3)	15.2 (9.1)	11.8 (7.7)	17.3 (7.9)
Fasting blood glucose, mean (SD), mmol/L	5.8 (1.6)	5.6 (1.5)	6.2 (1.8)	5.1 (0.5)	7.5 (2.0)
Hemoglobin A1c, mean (SD), %	6.3 (1.0)	6.2 (1.0)	6.3 (1.0)	5.7 (0.3)	7.5 (0.7)
HOMA2-IR	1.8 (0.7)	1.6 (0.7)	2.0 (0.4)	1.6 (0.5)	2.2 (0.8)
HOMA2-%β	121.0 (60.1)	125.6 (64.6)	113.7 (58.5)	130.8 (27.8)	99.1 (107.1)

Abbreviations: HOMA2, Homeostasis Model Assessment 2; IR, insulin resistance; SD, standard deviation; %β, beta-cell function.

Supplementary Table S3. *The detected reads and quality control of sequencing.*

Sample	Reads[a]	Bases[b], G	Q20[c], %	Q30[d], %	GC content, %
Sample1_baseline	9,960,089	0.498	99.28	97.49	53.10
Sample1_three months	1,057,1097	0.529	99.49	97.99	53.55
Sample1_six months	1,098,1263	0.549	99.54	98.22	53.61
Sample2_baseline	11,335,308	0.567	99.54	98.22	53.46
Sample2_three months	11,645,632	0.582	99.57	98.41	53.50
Sample2_six months	11,483,971	0.574	99.23	97.06	53.53
Sample3_baseline	12,063,005	0.603	99.61	98.40	53.77
Sample3_three months	13,957,910	0.698	99.35	97.81	53.05
Sample3_six months	11,327,730	0.566	99.34	97.78	52.94
Sample4_baseline	10,585,250	0.529	99.55	98.22	53.52
Sample4_three months	11,438,611	0.572	99.54	98.26	53.72
Sample4_six months	10,523,325	0.526	99.61	98.49	53.87
Sample5_baseline	11,148,967	0.557	98.88	96.57	54.61
Sample5_three months	10,975,332	0.549	97.49	93.32	51.32
Sample5_six months	11,148,630	0.557	99.54	98.22	53.88
Sample6_baseline	12,147,602	0.607	99.35	97.83	53.76
Sample6_three months	12,538,937	0.627	99.36	97.87	54.12
Sample6_six months	13,035,137	0.652	99.33	97.75	53.93
Sample7_baseline	13,592,944	0.680	99.35	97.79	53.80
Sample7_three months	10,598,328	0.530	99.28	97.54	53.77
Sample7_six months	10,297,237	0.515	99.56	98.36	53.42
Sample8_baseline	10,045,003	0.502	99.52	97.92	53.81
Sample8_three months	13,546,582	0.677	99.62	98.60	53.96
Sample8_six months	11,259,383	0.563	99.50	98.08	53.64
Sample9_baseline	11,629,044	0.581	99.62	98.73	54.11
Sample9_three months	12,429,427	0.621	99.58	98.56	53.61
Sample9_six months	15,684,982	0.784	99.60	98.55	53.40
Sample10_baseline	13,053,147	0.653	99.61	98.63	53.59
Sample10_three months	10,667,226	0.533	99.58	98.47	53.87
Sample10_six months	12,056,530	0.603	99.59	98.55	53.69
Sample11_baseline	14,413,220	0.721	99.55	98.25	53.84
Sample11_three months	11,291,928	0.565	99.60	98.57	53.74
Sample11_six months	12,357,112	0.618	99.41	98.01	53.76
Sample12_baseline	11,611,268	0.581	99.58	98.48	53.60
Sample12_three months	10,977,235	0.549	99.43	97.85	53.71
Sample12_six months	11,954,006	0.598	99.41	97.78	53.58
Sample13_baseline	11,929,639	0.596	99.44	97.86	53.99
Sample13_three months	12,196,726	0.610	99.44	97.76	53.90
Sample13_six months	11,078,323	0.554	99.41	97.80	53.15
Sample14_baseline	12,306,906	0.615	99.20	97.26	54.02
Sample14_three months	12,245,469	0.612	99.44	97.97	53.68
Sample14_six months	12,405,467	0.620	99.42	97.83	53.68
Sample15_baseline	14,582,201	0.729	99.47	98.08	54.08
Sample15_three months	13,235,303	0.662	99.45	97.93	54.16
Sample15_six months	11,328,953	0.566	99.21	97.26	53.89
Sample16_baseline	13,741,085	0.687	99.37	97.92	54.02
Sample16_three months	10,319,243	0.516	99.57	98.27	53.42
Sample16_six months	15,517,118	0.776	99.25	97.47	53.41

[a]Indicating the total number of sequence reads generated for each sample.

[b]Representing the total number of bases sequenced, given in gigabases (G).

[c]The percentage of bases with a quality score of 20 or higher, indicating a 99% probability that the base call is correct.

[d]The percentage of bases with a quality score of 30 or higher, indicating a 99.9% probability of accuracy.

***Supplementary Table S4**. Differentially expressed exosomal microRNAs in remission* vs. *non-remission of type 2 diabetes.*

MicroRNA	Coefficient[a]	Lower 95% CI	Upper 95% CI	FDR-adjusted *P*
miR-1185-1-3p	33.70	33.70	33.71	<0.001
miR-133a-3p	-2.87	-2.88	-2.85	<0.001
miR-15b-3p	-29.11	-29.30	-28.91	<0.001
miR-16-2-3p	-6.53	-6.54	-6.52	<0.001
miR-181d-5p	17.95	17.91	17.99	<0.001
miR-26a-5p	21.68	6.52	36.83	0.005
miR-3177-3p	-141.72	-141.73	-141.72	<0.001
miR-369-5p	0.45	0.41	0.49	<0.001
miR-3913-5p	-55.73	-80.45	-31.01	<0.001
miR-412-5p	-34.69	-34.70	-34.69	0.002
miR-452-5p	14.04	14.04	14.05	<0.001
miR-494-3p	26.95	26.94	26.96	<0.001
miR-495-3p	18.60	18.59	18.61	<0.001
miR-548f-3p	21.55	21.54	21.57	<0.001
miR-6852-5p	-113.70	-150.77	-76.64	<0.001
miR-760	-40.18	-62.54	-17.75	<0.001
miR-92b-3p	-3.24	-3.25	-3.23	<0.001
miR-98-5p	31.11	31.09	31.14	<0.001

Abbreviations: CI, confidence interval; FDR, false discovery rate.

[a]Coefficients were from the generalized linear mixed model with remission or not as outcome, adjusted for fixed effects including age, sex, duration of type 2 diabetes, and baseline body measure index, with each patient treated as a random effect.

Supplementary Table S5. Differentially expressed miRNAs in weight loss ≥10% vs. <10% groups.

MicroRNA	Coefficient[a]	Lower 95% CI	Upper 95% CI	FDR-adjusted *P*
miR-1185-1-3p	-19.02	-26.01	-12.03	<0.001
miR-133a-3p	-6.69	-6.69	-6.69	<0.001
miR-15b-3p	14.61	14.60	14.62	<0.001
miR-16-2-3p	5.92	5.90	5.95	<0.001
miR-181d-5p	-4.07	-4.08	-4.06	<0.001
miR-26a-5p	-20.04	-20.07	-20.01	<0.001
miR-3177-3p	0.82	0.82	0.83	<0.001
miR-369-5p	19.41	19.41	19.41	<0.001
miR-3913-5p	-10.67	-18.94	-2.40	0.04
miR-412-5p	-10.01	-10.01	-10.00	<0.001
miR-452-5p	1.311	1.30	1.32	<0.001
miR-494-3p	-12.25	-17.76	-6.74	<0.001
miR-495-3p	-10.48	-10.49	-10.46	<0.001
miR-548f-3p	0.54	0.53	0.55	<0.001
miR-6852-5p	5.57	5.56	5.57	<0.001
miR-760	6.61	6.61	6.62	<0.001
miR-92b-3p	-0.06	-0.06	-0.05	<0.001
miR-98-5p	-1.35	-1.36	-1.34	<0.001

Abbreviations: CI, confidence interval; FDR, false discovery rate.

[a]Coefficients were from the generalized linear mixed model with weight loss ≥10% or not as outcome, adjusted for fixed effects including age, sex, duration of type 2 diabetes, and baseline body measure index, with each patient treated as a random effect.

Supplementary Table S6. *Differential miRNA expression profiles in individuals with and without family history of type 2 diabetes.*

**miRNA**	**With family history (mean ± SD)**	**Without family history (mean ± SD)**	***P* value**	**FDR-adjusted *P***	**Fold change**
miR-495-3p	5.42 ± 1.20	6.61 ± 1.31	<0.01	0.02	0.82
miR-494-3p	3.92 ± 1.50	5.05 ± 1.27	0.01	0.08	0.78
miR-1185-1-3p	3.02 ± 0.98	3.90 ± 1.35	0.02	0.11	0.77
miR-369-5p	3.86 ± 1.10	4.63 ± 1.15	0.03	0.11	0.83
miR-760	4.29 ± 1.30	3.39 ± 1.16	0.04	0.13	1.27
miR-548f-3p	3.04 ± 0.90	3.50 ± 0.88	0.10	0.30	0.87
miR-26a-5p	15.48 ± 1.27	15.97 ± 1.09	0.12	0.32	0.97
miR-412-5p	2.39 ± 0.73	2.91 ± 1.07	0.14	0.32	0.82
miR-92b-3p	3.37 ± 0.86	3.08 ± 0.88	0.18	0.35	1.10
miR-16-2-3p	7.38 ± 1.92	8.07 ± 0.85	0.20	0.36	0.91
miR-6852-5p	7.49 ± 1.52	7.42 ± 0.82	0.25	0.39	1.01
miR-181d-5p	5.68 ± 1.40	6.13 ± 0.74	0.26	0.39	0.93
miR-15b-3p	3.92 ± 1.19	4.28 ± 1.16	0.30	0.42	0.92
miR-98-5p	8.74 ± 1.78	9.14 ± 1.10	0.38	0.46	0.96
miR-3913-5p	4.20 ± 1.15	4.52 ± 0.87	0.40	0.46	0.93
miR-452-5p	4.49 ± 1.30	4.34 ± 1.07	0.41	0.46	1.04
miR-3177-3p	5.04 ± 1.17	5.16 ± 0.77	0.57	0.61	0.98
miR-133a-3p	4.02 ± 1.08	3.90 ± 1.19	0.70	0.70	1.03

Abbreviations: FDR, false discovery rate; SD, standard deviation.

Supplementary Table S7*. Differentially expressed miRNAs targets that strong validated.*

MicroRNA	Target	Support	Source	Experiment(s)	Reference PMID
miR-133a-3p	MCL1	Functional MTI	MIRT052648	Luciferase reporter assay, Western blot	23756231
miR-133a-3p	MCL1	Functional MTI	MIRT052648	ChIP-seq, Luciferase reporter assay, qRT-PCR, Western blot	26276722
miR-133a-3p	IGF1R	Functional MTI	MIRT054310	Luciferase reporter assay, qRT-PCR, Western blot	25780292
miR-133a-3p	IGF1R	Functional MTI	MIRT054310	Luciferase reporter assay, qRT-PCR, Western blot	24127040
miR-133a-3p	IGF1	Functional MTI	MIRT731340	Luciferase reporter assay	26845446
miR-133a-3p	ZEB1	Functional MTI	MIRT731591	Luciferase reporter assay	26902265
miR-133a-3p	MEG3	Functional MTI	MIRT733021	qRT-PCR, Western blot	28320084
miR-15b-3p	IGF1R	Functional MTI	MIRT731817	Luciferase reporter assay, Western blot	27896672
miR-15b-3p	RECK	Functional MTI	MIRT731853	Western blot	27530410
miR-181d-5p	BCL2	Functional MTI	MIRT003498	Luciferase reporter assay, qRT-PCR, Western blot, Reporter assay;Western blot;Other	20162574
miR-181d-5p	BCL2	Functional MTI	MIRT003498	FACS, Immunohistochemistry, Luciferase reporter assay, qRT-PCR, Western blot	22207524
miR-181d-5p	MEG3	Functional MTI	MIRT731767	Luciferase reporter assay, qRT-PCR, Western blot	26253106
miR-181d-5p	MALT1	Functional MTI	MIRT734325	Luciferase reporter assay, Microarray, Next Generation Sequencing (NGS), qRT-PCR, Western blot	28286260
miR-26a-5p	HMGA2	Functional MTI	MIRT000109	Luciferase reporter assay	17563749
miR-26a-5p	HMGA2	Functional MTI	MIRT000109	Luciferase reporter assay, qRT-PCR, Western blot	22139073
miR-26a-5p	HMGA2	Functional MTI	MIRT000109	Luciferase reporter assay, qRT-PCR, Western blot	24853416
miR-26a-5p	HMGA2	Functional MTI	MIRT000109	Luciferase reporter assay	24682444
miR-26a-5p	HMGA2	Functional MTI	MIRT000109	Luciferase reporter assay, qRT-PCR, Western blot	26492332
miR-26a-5p	CCND2	Functional MTI	MIRT000112	Luciferase reporter assay, Western blot	19524505
miR-26a-5p	PTEN	Functional MTI	MIRT001095	Western blot, Luciferase reporter assay	19487573

miR-26a-5p	PTEN	Functional MTI	MIRT001095	Luciferase reporter assay, Western blot	20216554
miR-26a-5p	PTEN	Functional MTI	MIRT001095	GFP reporter assay, Luciferase reporter assay, Western blot	20080666
miR-26a-5p	PTEN	Functional MTI	MIRT001095	Luciferase reporter assay, Western blot	22885155
miR-26a-5p	PTEN	Functional MTI	MIRT001095	Luciferase reporter assay	24140063
miR-26a-5p	PTEN	Functional MTI	MIRT001095	qRT-PCR, Luciferase reporter assay, Western blot	25361012
miR-26a-5p	PTEN	Functional MTI	MIRT001095	Immunohistochemistry, Luciferase reporter assay, qRT-PCR, Western blot	28242043
miR-26a-5p	EZH2	Functional MTI	MIRT001771	Luciferase reporter assay, Western blot	18713946
miR-26a-5p	EZH2	Functional MTI	MIRT001771	Luciferase reporter assay, qRT-PCR, Western blot	20478051
miR-26a-5p	EZH2	Functional MTI	MIRT001771	Immunohistochemistry, qRT-PCR, Western blot	21199804
miR-26a-5p	EZH2	Functional MTI	MIRT001771	Immunohistochemistry, Luciferase reporter assay, qRT-PCR, Western blot	20952513
miR-26a-5p	EZH2	Functional MTI	MIRT001771	Immunoblot, Immunohistochemistry, Luciferase reporter assay, Microarray, qRT-PCR	21368858
miR-26a-5p	EZH2	Functional MTI	MIRT001771	Luciferase reporter assay, Microarray, qRT-PCR, Western blot	21829663
miR-26a-5p	EZH2	Functional MTI	MIRT001771	Reporter assay	18281287
miR-26a-5p	EZH2	Functional MTI	MIRT001771	Immunohistochemistry, qRT-PCR, Western blot	23599767
miR-26a-5p	EZH2	Functional MTI	MIRT001771	Luciferase reporter assay, qRT-PCR, Western blot	28214878
miR-26a-5p	EZH2	Functional MTI	MIRT001771	qRT-PCR, Western blot	25611389
miR-26a-5p	EZH2	Functional MTI	MIRT001771	Luciferase reporter assay, qRT-PCR, Western blot	26733151
miR-26a-5p	EZH2	Functional MTI	MIRT001771	Luciferase reporter assay, qRT-PCR, Western blot, ChIP	26781064
miR-26a-5p	EZH2	Functional MTI	MIRT001771	Luciferase reporter assay	26587974
miR-26a-5p	CDK6	Functional MTI	MIRT006306	Luciferase reporter assay, Western blot	22210897
miR-26a-5p	IL6	Functional MTI	MIRT007374	Luciferase reporter assay	23389848
miR-26a-5p	MCL1	Functional MTI	MIRT052649	Western blot	23750239

miR-26a-5p	DNMT3B	Functional MTI	MIRT118410	Luciferase reporter assay, Microarray, qRT-PCR, Western blot	24343426
miR-26a-5p	NRAS	Functional MTI	MIRT732062	Luciferase reporter assay, qRT-PCR, Western blot	26458859
miR-26a-5p	E2F2	Functional MTI	MIRT732063	Luciferase reporter assay, qRT-PCR, Western blot	26458859
miR-26a-5p	MALT1	Functional MTI	MIRT732676	Western blot	27025651
miR-26a-5p	IGF1	Functional MTI	MIRT734538	Luciferase reporter assay, qRT-PCR, Western blot	27468358
miR-369-5p	DNMT3B	Functional MTI	MIRT005961	Luciferase reporter assay, Reporter assay;Western blot;qRT-PCR	21660946
miR-452-5p	BMI1	Functional MTI	MIRT437780	Immunofluorescence, Western blot	23695168
miR-494-3p	PTEN	Functional MTI	MIRT001209	qRT-PCR, Luciferase reporter assay, Western blot	20006626
miR-494-3p	PTEN	Functional MTI	MIRT001209	Flow, Luciferase reporter assay, Microarray, qRT-PCR, Western blot	22544933
miR-494-3p	PTEN	Functional MTI	MIRT001209	Immunofluorescence, Immunohistochemistry, qRT-PCR, Western blot	25662849
miR-494-3p	CDK6	Functional MTI	MIRT006112	Luciferase reporter assay, Microarray, qRT-PCR, Western blot	21809359
miR-494-3p	BMI1	Functional MTI	MIRT053512	Luciferase reporter assay, Microarray, qRT-PCR, Western blot	23105110
miR-494-3p	BMI1	Functional MTI	MIRT053512	Luciferase reporter assay, qRT-PCR, Western blot	27399693
miR-494-3p	ZEB1	Functional MTI	MIRT053515	Luciferase reporter assay, Microarray, qRT-PCR, Western blot	23105110
miR-494-3p	MYC	Functional MTI	MIRT053756	Luciferase reporter assay, qRT-PCR, Western blot	24612089
miR-494-3p	BCL2	Functional MTI	MIRT438772	qRT-PCR, Western blot	24960059
miR-494-3p	IGF1R	Functional MTI	MIRT536716	Luciferase reporter assay, qRT-PCR, Western blot	27735036
miR-494-3p	IGF1R	Functional MTI	MIRT536716	Luciferase reporter assay, Western blot	26695144
miR-494-3p	AKT1	Functional MTI	MIRT734751	Immunofluorescence, Immunohistochemistry, qRT-PCR, Western blot	25662849

miR-495-3p	PBX3	Functional MTI	MIRT007194	Immunoblot, Immunohistochemistry, Luciferase reporter assay, Microarray, qRT-PCR, Western blot	23132946
miR-495-3p	BMI1	Functional MTI	MIRT053513	Luciferase reporter assay, Microarray, qRT-PCR, Western blot	23105110
miR-495-3p	BMI1	Functional MTI	MIRT053513	Luciferase reporter assay, Western blot, Immunohistochemistry	26020378
miR-495-3p	BMI1	Functional MTI	MIRT053513	Luciferase reporter assay, qRT-PCR, Western blot	28628915
miR-495-3p	CCL2	Functional MTI	MIRT731131	ELISA, qRT-PCR, Luciferase reporter assay, Western blot	25466836
miR-495-3p	AKT1	Functional MTI	MIRT731805	Luciferase reporter assay, qRT-PCR, Western blot	27323412
miR-495-3p	HMGA2	Functional MTI	MIRT734067	Luciferase reporter assay, qRT-PCR, Western blot	28159956
miR-92b-3p	PTEN	Functional MTI	MIRT438261	Luciferase reporter assay	24099768
miR-92b-3p	PTEN	Functional MTI	MIRT438261	Luciferase reporter assay, Western blot	28337377
miR-92b-3p	RECK	Functional MTI	MIRT438481	Luciferase reporter assay	24162673
miR-92b-3p	RECK	Functional MTI	MIRT438481	Luciferase reporter assay, Western blot	26993249
miR-98-5p	E2F2	Functional MTI	MIRT001126	Northern blot, Western blot, qRT-PCR, ChIP, Luciferase reporter assay	19528081
miR-98-5p	HMGA2	Functional MTI	MIRT002999	Northern blot, qRT-PCR, Western blot;Other	17222355
miR-98-5p	HMGA2	Functional MTI	MIRT002999	Luciferase reporter assay, Western blot	24392454
miR-98-5p	MYC	Functional MTI	MIRT003438	Luciferase reporter assay, Northern blot, qRT-PCR, ChIP	19528081
miR-98-5p	EZH2	Functional MTI	MIRT005721	Immunoblot, Immunohistochemistry, Luciferase reporter assay, Microarray, qRT-PCR	21368858
miR-98-5p	EZH2	Functional MTI	MIRT005721	Luciferase reporter assay, qRT-PCR, Western blot	27890434
miR-98-5p	IL6	Functional MTI	MIRT027449	Luciferase reporter assay, qRT-PCR, Western blot	26587789
miR-98-5p	NRAS	Functional MTI	MIRT027862	Immunofluorescence, In situ hybridization, Microarray, qRT-PCR, Western blot	26244871

miR-98-5p	CCND2	Functional MTI	MIRT134736	GFP reporter assay	26840039
miR-98-5p	IGF1	Functional MTI	MIRT732227	Luciferase reporter assay, qRT-PCR, Western blot	26722410
miR-98-5p	PBX3	Functional MTI	MIRT732086	Luciferase reporter assay, qRT-PCR, Western blot	28124208
miR-98-5p	CCL2	Functional MTI	MIRT732865	ELISA, Luciferase reporter assay, qRT-PCR, Western blot	26126865
miR-98-5p	BCL2	Functional MTI	MIRT735452	Luciferase reporter assay, Western blot	27422937

Supplementary Table S8*. Differentially expressed miRNAs targets in Kyoto Encyclopedia of Genes and Genomes.*

Category	Subcategory	ID	Description	Gene ratio	FDR-adjusted *P*	GeneID	Count	Contributing miRNAs	miRNA count
Environmental Information Processing	Signal transduction	hsa04151	PI3K-Akt signaling pathway	0.0271	6.06E-09	MCL1/IGF1R/IGF1/BCL2/CCND2/PTEN/CDK6/IL6/NRAS/MYC/AKT1	11	hsa-miR-133a-3p; hsa-miR-15b-3p; hsa-miR-181d-5p; hsa-miR-26a-5p; hsa-miR-494-3p; hsa-miR-495-3p; hsa-miR-92b-3p; hsa-miR-98-5p	8
Human Diseases	Drug resistance: antineoplastic	hsa01521	EGFR tyrosine kinase inhibitor resistance	0.0172	9.17E-09	IGF1R/IGF1/BCL2/PTEN/IL6/NRAS/AKT1	7	hsa-miR-133a-3p; hsa-miR-15b-3p; hsa-miR-181d-5p; hsa-miR-26a-5p; hsa-miR-494-3p; hsa-miR-495-3p; hsa-miR-92b-3p; hsa-miR-98-5p	8
Cellular Processes	Cell growth and death	hsa04218	Cellular senescence	0.0197	2.47E-08	CCND2/PTEN/CDK6/IL6/NRAS/E2F2/MYC/AKT1	8	hsa-miR-26a-5p; hsa-miR-494-3p; hsa-miR-495-3p; hsa-miR-92b-3p; hsa-miR-98-5p	5
Environmental Information Processing	Signal transduction	hsa04068	FoxO signaling pathway	0.0172	2.33E-07	IGF1R/IGF1/CCND2/PTEN/IL6/NRAS/AKT1	7	hsa-miR-133a-3p; hsa-miR-15b-3p; hsa-miR-26a-5p; hsa-miR-494-3p; hsa-miR-495-3p; hsa-miR-92b-3p; hsa-miR-98-5p	7
Human Diseases	Drug resistance: antineoplastic	hsa01522	Endocrine resistance	0.0148	9.93E-07	IGF1R/IGF1/BCL2/NRAS/E2F2/AKT1	6	hsa-miR-133a-3p; hsa-miR-15b-3p; hsa-miR-181d-5p; hsa-miR-26a-5p; hsa-miR-494-3p; hsa-miR-495-3p; hsa-miR-98-5p	7
Cellular Processes	Cell growth and death	hsa04115	p53 signaling pathway	0.0123	6.67E-06	IGF1/BCL2/CCND2/PTEN/CDK6	5	hsa-miR-133a-3p; hsa-miR-181d-5p; hsa-miR-26a-5p; hsa-miR-494-3p; hsa-miR-92b-3p; hsa-miR-98-5p	6

Cellular Processes	Cellular community - eukaryotes	hsa04550	Signaling pathways regulating pluripotency of stem cells	0.0148	6.68E-06	IGF1R/IGF1/NRAS/BMI1/MYC/AKT1	6	hsa-miR-133a-3p; hsa-miR-15b-3p; hsa-miR-26a-5p; hsa-miR-452-5p; hsa-miR-494-3p; hsa-miR-495-3p; hsa-miR-98-5p	7
Environmental Information Processing	Signal transduction	hsa04630	JAK-STAT signaling pathway	0.0148	1.41E-05	MCL1/BCL2/CCND2/IL6/MYC/AKT1	6	hsa-miR-133a-3p; hsa-miR-181d-5p; hsa-miR-26a-5p; hsa-miR-494-3p; hsa-miR-495-3p; hsa-miR-98-5p	6
Human Diseases	Endocrine and metabolic disease	hsa04933	AGE-RAGE signaling pathway in diabetic complications	0.0123	2.27E-05	BCL2/IL6/NRAS/AKT1/CCL2	5	hsa-miR-181d-5p; hsa-miR-26a-5p; hsa-miR-494-3p; hsa-miR-495-3p; hsa-miR-98-5p	5
Human Diseases	Infectious disease: viral	hsa05167	Kaposi sarcoma-associated herpesvirus infection	0.0148	3.04E-05	CDK6/IL6/NRAS/E2F2/MYC/AKT1	6	hsa-miR-26a-5p; hsa-miR-494-3p; hsa-miR-495-3p; hsa-miR-98-5p	4
Cellular Processes	Cellular community - eukaryotes	hsa04510	Focal adhesion	0.0148	3.57E-05	IGF1R/IGF1/BCL2/CCND2/PTEN/AKT1	6	hsa-miR-133a-3p; hsa-miR-15b-3p; hsa-miR-181d-5p; hsa-miR-26a-5p; hsa-miR-494-3p; hsa-miR-495-3p; hsa-miR-92b-3p; hsa-miR-98-5p	8
Organismal Systems	Aging	hsa04213	Longevity regulating pathway - multiple species	0.0098	7.19E-05	IGF1R/IGF1/NRAS/AKT1	4	hsa-miR-133a-3p; hsa-miR-15b-3p; hsa-miR-26a-5p; hsa-miR-494-3p; hsa-miR-495-3p; hsa-miR-98-5p	6
Environmental Information Processing	Signal transduction	hsa04150	mTOR signaling pathway	0.0123	0.000139	IGF1R/IGF1/PTEN/NRAS/AKT1	5	hsa-miR-133a-3p; hsa-miR-15b-3p; hsa-miR-26a-5p; hsa-miR-494-3p; hsa-miR-	7

								495-3p; hsa-miR-92b-3p; hsa-miR-98-5p	
Cellular Processes	Transport and catabolism	hsa04140	Autophagy - animal	0.0123	0.000181	IGF1R/BCL2/PTEN/NRAS/AKT1	5	hsa-miR-133a-3p; hsa-miR-15b-3p; hsa-miR-181d-5p; hsa-miR-26a-5p; hsa-miR-494-3p; hsa-miR-495-3p; hsa-miR-92b-3p; hsa-miR-98-5p	8
Organismal Systems	Aging	hsa04211	Longevity regulating pathway	0.0098	0.000237	IGF1R/IGF1/NRAS/AKT1	4	hsa-miR-133a-3p; hsa-miR-15b-3p; hsa-miR-26a-5p; hsa-miR-494-3p; hsa-miR-495-3p; hsa-miR-98-5p	6
Organismal Systems	Immune system	hsa04625	C-type lectin receptor signaling pathway	0.0098	0.00041	MALT1/IL6/NRAS/AKT1	4	hsa-miR-181d-5p; hsa-miR-26a-5p; hsa-miR-494-3p; hsa-miR-495-3p; hsa-miR-98-5p	5
Human Diseases	Cardiovascular disease	hsa05417	Lipid and atherosclerosis	0.0123	0.000496	BCL2/IL6/NRAS/AKT1/CCL2	5	hsa-miR-181d-5p; hsa-miR-26a-5p; hsa-miR-494-3p; hsa-miR-495-3p; hsa-miR-98-5p	5
Environmental Information Processing	Signal transduction	hsa04071	Sphingolipid signaling pathway	0.0098	0.00068	BCL2/PTEN/NRAS/AKT1	4	hsa-miR-181d-5p; hsa-miR-26a-5p; hsa-miR-494-3p; hsa-miR-495-3p; hsa-miR-92b-3p; hsa-miR-98-5p	6
Cellular Processes	Cell growth and death	hsa04110	Cell cycle	0.0098	0.001704	CCND2/CDK6/E2F2/MYC	4	hsa-miR-26a-5p; hsa-miR-494-3p; hsa-miR-98-5p	3
Environmental Information Processing	Signal transduction	hsa04010	MAPK signaling pathway	0.0123	0.001986	IGF1R/IGF1/NRAS/MYC/AKT1	5	hsa-miR-133a-3p; hsa-miR-15b-3p; hsa-miR-26a-5p; hsa-miR-494-3p; hsa-miR-495-3p; hsa-miR-98-5p	6

Abbreviation: FDR, false discovery rate.

Supplementary Table S9*. Differentially expressed miRNAs targets in Gene Ontology.*

ONTOLOGY	ID	Description	Gene ratio	FDR-adjusted *P*	geneID	Count	Contributing miRNAs	miRNA count
BP	GO:0051347	positive regulation of transferase activity	10/22	8.39E-09	IGF1R/IGF1/MALT1/HMGA2/CCND2/PTEN/EZH2/BMI1/MYC/AKT1	10	hsa-miR-133a-3p; hsa-miR-15b-3p; hsa-miR-181d-5p; hsa-miR-26a-5p; hsa-miR-452-5p; hsa-miR-494-3p; hsa-miR-495-3p; hsa-miR-92b-3p; hsa-miR-98-5p	9
BP	GO:0000082	G1/S transition of mitotic cell cycle	8/22	1.25E-07	BCL2/CCND2/PTEN/EZH2/CDK6/MYC/AKT1/CCL2	8	hsa-miR-181d-5p; hsa-miR-26a-5p; hsa-miR-494-3p; hsa-miR-495-3p; hsa-miR-92b-3p; hsa-miR-98-5p	6
BP	GO:0044843	cell cycle G1/S phase transition	8/22	2.05E-07	BCL2/CCND2/PTEN/EZH2/CDK6/MYC/AKT1/CCL2	8	hsa-miR-181d-5p; hsa-miR-26a-5p; hsa-miR-494-3p; hsa-miR-495-3p; hsa-miR-92b-3p; hsa-miR-98-5p	6
BP	GO:0033674	positive regulation of kinase activity	7/22	1.08E-05	IGF1R/IGF1/MALT1/HMGA2/CCND2/EZH2/AKT1	7	hsa-miR-133a-3p; hsa-miR-15b-3p; hsa-miR-181d-5p; hsa-miR-26a-5p; hsa-miR-494-3p; hsa-miR-495-3p; hsa-miR-98-5p	7
BP	GO:0050673	epithelial cell proliferation	8/22	1.08E-05	IGF1/ZEB1/CDK6/IL6/NRAS/MYC/AKT1/CCL2	8	hsa-miR-133a-3p; hsa-miR-26a-5p; hsa-miR-494-3p; hsa-miR-495-3p; hsa-miR-98-5p	5
BP	GO:1903131	mononuclear cell differentiation	8/22	1.08E-05	ZEB1/BCL2/MALT1/EZH2/CDK6/IL6/BMI1/MYC	8	hsa-miR-133a-3p; hsa-miR-181d-5p; hsa-miR-26a-5p; hsa-miR-452-5p; hsa-miR-494-3p; hsa-miR-495-3p; hsa-miR-98-5p	7
BP	GO:2000045	regulation of G1/S transition	6/22	1.23E-05	BCL2/CCND2/PTEN/EZH2/AKT1/CCL2	6	hsa-miR-181d-5p; hsa-miR-26a-5p; hsa-miR-494-3p; hsa-	6

		of mitotic cell cycle					miR-495-3p; hsa-miR-92b-3p; hsa-miR-98-5p	
BP	GO:0051251	positive regulation of lymphocyte activation	7/22	1.25E-05	IGF1/BCL2/MALT1/IL6/BMI1/AKT1/CCL2	7	hsa-miR-133a-3p; hsa-miR-181d-5p; hsa-miR-26a-5p; hsa-miR-452-5p; hsa-miR-494-3p; hsa-miR-495-3p; hsa-miR-98-5p	7
BP	GO:0060485	mesenchyme development	7/22	1.33E-05	IGF1/BCL2/HMGA2/PTEN/EZH2/IL6/MYC	7	hsa-miR-133a-3p; hsa-miR-181d-5p; hsa-miR-26a-5p; hsa-miR-494-3p; hsa-miR-495-3p; hsa-miR-92b-3p; hsa-miR-98-5p	7
BP	GO:1902806	regulation of cell cycle G1/S phase transition	6/22	1.7E-05	BCL2/CCND2/PTEN/EZH2/AKT1/CCL2	6	hsa-miR-181d-5p; hsa-miR-26a-5p; hsa-miR-494-3p; hsa-miR-495-3p; hsa-miR-92b-3p; hsa-miR-98-5p	6
CC	GO:0097136	Bcl-2 family protein complex	2/22	0.003457	MCL1/BCL2	2	hsa-miR-133a-3p; hsa-miR-181d-5p; hsa-miR-26a-5p; hsa-miR-494-3p; hsa-miR-98-5p	5
CC	GO:0000792	heterochromatin	3/22	0.003457	HMGA2/EZH2/BMI1	3	hsa-miR-26a-5p; hsa-miR-452-5p; hsa-miR-494-3p; hsa-miR-495-3p; hsa-miR-98-5p	5
CC	GO:1902911	protein kinase complex	3/22	0.014172	IGF1R/CCND2/CDK6	3	hsa-miR-133a-3p; hsa-miR-15b-3p; hsa-miR-26a-5p; hsa-miR-494-3p; hsa-miR-98-5p	5
CC	GO:0043209	myelin sheath	2/22	0.019423	BCL2/PTEN	2	hsa-miR-181d-5p; hsa-miR-26a-5p; hsa-miR-494-3p; hsa-miR-92b-3p; hsa-miR-98-5p	5
CC	GO:0031519	PcG protein complex	2/22	0.019423	EZH2/BMI1	2	hsa-miR-26a-5p; hsa-miR-452-5p; hsa-miR-494-3p; hsa-miR-495-3p; hsa-miR-98-5p	5
MF	GO:0070513	death domain binding	2/21	0.009503	MCL1/BCL2	2	hsa-miR-133a-3p; hsa-miR-181d-5p; hsa-miR-26a-5p; hsa-miR-494-3p; hsa-miR-98-5p	5

MF	GO:0005158	insulin receptor binding	2/21	0.019807	IGF1R/IGF1	2	hsa-miR-133a-3p; hsa-miR-15b-3p; hsa-miR-26a-5p; hsa-miR-494-3p; hsa-miR-98-5p	5
MF	GO:0002020	protease binding	3/21	0.024081	BCL2/MALT1/PTEN	3	hsa-miR-181d-5p; hsa-miR-26a-5p; hsa-miR-494-3p; hsa-miR-92b-3p; hsa-miR-98-5p	5
MF	GO:0140318	protein transporter activity	2/21	0.02913	MCL1/IGF1R	2	hsa-miR-133a-3p; hsa-miR-15b-3p; hsa-miR-26a-5p; hsa-miR-494-3p	4
MF	GO:0001046	core promoter sequence-specific DNA binding	2/21	0.02913	EZH2/MYC	2	hsa-miR-26a-5p; hsa-miR-494-3p; hsa-miR-98-5p	3
MF	GO:0003714	transcription corepressor activity	3/21	0.03286	HMGA2/EZH2/DNMT3B	3	hsa-miR-26a-5p; hsa-miR-369-5p; hsa-miR-495-3p; hsa-miR-98-5p	4
MF	GO:0070888	E-box binding	2/21	0.040872	ZEB1/MYC	2	hsa-miR-133a-3p; hsa-miR-494-3p; hsa-miR-98-5p	3
MF	GO:1990841	promoter-specific chromatin binding	2/21	0.04462	EZH2/BMI1	2	hsa-miR-26a-5p; hsa-miR-452-5p; hsa-miR-494-3p; hsa-miR-495-3p; hsa-miR-98-5p	5

Abbreviations: BP, biological process; CC, cellular component; FDR, false discovery rate; MF, molecular function.
